# Dynamics of Gut Bacteria Across Different Zooplankton Genera in the Baltic Sea

**DOI:** 10.1007/s00248-024-02362-7

**Published:** 2024-02-26

**Authors:** Tianshuo Xu, Andreas Novotny, Sara Zamora-Terol, Peter A. Hambäck, Monika Winder

**Affiliations:** 1 Department of Ecology Environment and Plant Sciences, Stockholm University, Stockholm, Sweden; 2 Present Address: Institute for the Oceans and Fisheries, University of British Columbia, Vancouver, Canada; 3 Present Address: Institute of Marine Research, Bergen, Norway

**Keywords:** Zooplankton, Gut microbiome, Temporal variability, Host specificity, Diet effects

## Abstract

In aquatic ecosystems, zooplankton-associated bacteria potentially have a great impact on the structure of ecosystems and trophic networks by providing various metabolic pathways and altering the ecological niche of host species. To understand the composition and drivers of zooplankton gut microbiota, we investigated the associated microbial communities of four zooplankton genera from different seasons in the Baltic Sea using the 16S rRNA gene. Among the 143 ASVs (amplified sequence variants) observed belonging to heterotrophic bacteria, 28 ASVs were shared across all zooplankton hosts over the season, and these shared core ASVs represented more than 25% and up to 60% of relative abundance in zooplankton hosts but were present at low relative abundance in the filtered water. Zooplankton host identity had stronger effects on bacterial composition than seasonal variation, with the composition of gut bacterial communities showing host-specific clustering patterns. Although bacterial compositions and dominating core bacteria were different between zooplankton hosts, higher gut bacteria diversity and more bacteria contributing to the temporal variation were found in *Temora* and *Pseudocalanus*, compared to *Acartia* and *Synchaeta*. Diet diatom and filamentous cyanobacteria negatively correlated with gut bacteria diversity, but the difference in diet composition did not explain the dissimilarity of gut bacteria composition, suggesting a general effect of diet on the inner conditions in the zooplankton gut. *Synchaeta* maintained high stability of gut bacterial communities with unexpectedly low bacteria-bacteria interactions as compared to the copepods, indicating host-specific regulation traits. Our results suggest that the patterns of gut bacteria dynamics are host-specific and the variability of gut bacteria is not only related to host taxonomy but also related to host behavior and life history traits.

**Supplementary Information:**

The online version contains supplementary material available at 10.1007/s00248-024-02362-7.

## Introduction

The microbial community associated to their hosts has multiple functions for ecological processes in both aquatic and terrestrial ecosystems [1–4]. For individual organisms, symbiotic bacteria provide additional trophic pathways, interact with the immune system, assimilate ambient nutrients, and mitigate pathogens or parasites [5, 6]. For ecosystems, bacterial symbiosis not only links to host niche occupation but also contributes to substance circulation at a large scale, such as nitrogen cycling and methane emission [7–9]. In aquatic ecosystems, bacterioplankton and zooplankton are key nodes in trophic networks that perform critical ecological functions by transforming, concentrating, and channeling carbon and essential nutrients across trophic levels [10, 11]. The concentration of bacteria associated to zooplankton is magnitudes higher than that of free-living bacterioplankton, creating bacterial activity hotspots that enhance nutrient cycling within the trophic network [12, 13]. Considering the significant biomass of global aquatic zooplankton and bacteria, the association of these organisms may significantly contribute to aquatic substance cycling [14, 15]. Determining the gut bacteria composition and dynamics is an important first step to understanding the role of zooplankton-associated bacteria in aquatic ecosystems. While the drivers and mechanisms of bacterial symbionts for plants and terrestrial animals are often described with theoretical frameworks [16, 17], drivers and spatiotemporal dynamics of the bacterial community associated to zooplankton hosts are not well known.

Consistent with other organisms, the functions of zooplankton-bacterial symbiont interactions are diverse. The gut microbiome may benefit their zooplankton hosts in the acclimation to different environments, breaking down indigestible diet compounds, degrading toxic substances, and combating parasitism [18–20]. Bacteria may also interact with the host immune system and even change the morphology of host guts, as shown for *Daphnia* and juvenile squid *Euprymna* [21, 22]. In return, hosts provide relatively stable gut conditions as a refuge for the symbionts, keeping bacteria away from stressful and fluctuating environmental factors [18]. The stability of the gut environment is host-specifically regulated, and the dynamics of symbiotic gut bacteria are controlled by a complex network of interactions [13, 18, 23]. These interactions involve bacteria traits related to the adaptive capacity to the host conditions, host-related mechanisms, such as habitat filtration of the immune systems or diet, and microbiome-related mechanisms, such as resource competition and antagonism [16, 24].

Although various factors regulate symbiont composition, the dynamics of zooplankton symbiotic bacteria follow a general metacommunity framework, corresponding to the bacteria recruitment process [13, 16–18, 25]. Seed bank and community assembly theory emphasize the importance of inoculation of background bacteria that shape the structure of the symbiotic community in various organisms [22, 26]. The inoculated bacteria subsequently experience screening of the host’s inner environment, interaction with local bacterial communities, and regulation of host metabolism. Hence, in both vertebrates and invertebrates, symbionts show environmental-specific (i.e., geological and seasonal) and host-specific (i.e., subpopulation and genotype) patterns [2, 19, 27–32]. In widely studied organisms, such as humans, hosts with similar traits tend to have bacterial communities with a similar set of dominant species [31, 33, 34]. Clustering of zooplankton-associated bacterial communities was also observed in field samples of zooplankton [27], but the zooplankton gut environment is likely less stable than the mammalian gut systems and is assumed to lack consistency of dominating bacteria species [28]. In addition, higher diversities of symbiotic bacteria correlate with higher resistance to disturbance because more diverse bacteria have more complete usage of niches in the gut environment [17, 34]. While these theories are important for understanding drivers affecting the dynamics of bacterial communities, they remain to be tested for specific zooplankton taxa.

To better understand the dynamics of zooplankton gut symbionts under the scope of general symbiosis ecology, we investigated bacterial compositions and diversity within a range of zooplankton hosts across a temporal gradient. We hypothesized that (H1) zooplankton-associated bacterial communities form clusters of different dominant species across host genera and that the variability of bacterial communities is host-specific. Because diet is one main factor shifting symbiotic bacteria composition in many organisms [19, 23, 31, 33, 35] and zooplankton have diverse feeding behaviors and diet compositions [36, 37], we assumed that (H2) the zooplankton bacteria dynamics are driven by feeding selectivity on phytoplankton. To test our hypotheses, the succession of and effects from prey selectivity for zooplankton-associated gut bacteria were tested for copepod and rotifer genera using Illumina sequencing of the *16S rRNA* gene in the Baltic Sea. For H1, we described the temporal variation of bacteria composition by illustrating host and seasonal clustering and alpha diversity for zooplankton-associated bacteria. Besides dominating bacteria, important bacteria during community fluctuations were determined by estimating the contribution of the different bacteria to the temporal variance of the symbiotic community in the different zooplankton hosts. We further examined bacteria correlation patterns to identify the stability of bacteria-bacteria interactions as an indication of distinct bacterial succession in the zooplankton host genera. For H2, we tested the effects of each diet component on the alpha diversity of bacterial communities and the correlation between the similarity of diet composition and the similarity of bacterial communities.

## Materials and Methods

### Sampling and Zooplankton Sorting

Samples were taken from the offshore monitoring station Landsort Deep (BY31, 58° 35′ N, 18° 14′ E) in the northern Baltic Sea proper in June and August 2017 and March 2018. Zooplankton were collected from vertical hauls at 0–30 m, 30–60 m, and 60–100 m with a 90-µm-WP2 net. Water samples were taken with 10-L Niskin bottles every 5 m from 0 to 30 m and every 10 m from 30 to 100 m depth, and equal volumes from each depth strata were pooled before further analysis. The samples were filtered with 25 mm filters placed in Swinnex holders (Merck/Millipore) with 0.2 and 2 µm polycarbonate and 20 µm nylon filters to separate planktonic communities. Filters were stored under − 80 °C until DNA extraction. Water samples filtered with different filtration sizes were combined in downstream analyses.

Four genera of zooplankton that were present throughout the sampling period were sorted by stereomicroscopy from the depth strata where they were most abundant, including adult stages of the copepods *Temora longicornis* and *Pseaudocalanus* spp. from 30 to 60 m depth, the copepod *Acartia* spp. and rotifer *Synchaeta baltica* from 0 to 30 m depth. Each individual zooplankton was rinsed with a bleach solution (~ 1%) and, if necessary, appendages were detached to remove potential ectosymbionts. Four to eight replicates with three to six individuals each were sorted per genus and month for DNA sequencing.

### DNA Extraction and Metabarcoding

DNA of filtered water samples was extracted with the DNeasy Plant Mini Kit (Qiagen), whereas zooplankton samples were lysed by bead beating using 1 mm glass beads, followed by DNA extraction with the QIAamp DNA Micro Kit (Qiagen). Universal primers 341F (CCTACGGGNGGCWGCAG) and 805R (GACTACHVGGGTATCTAATCC) were used for PCR-amplification of the V3-V4 region of the *16S rRNA* gene. The PCR amplicon libraries were sequenced with MiSeq (MSC 2.5.0.5/RTA 1.18.54) pair-end set-up (2 × 300 bp, v.3, Illumina, San Diego, California). A detailed description of sampling and metabarcoding analysis is provided by Zamora-Terol et al. (2020) and Novotny et al. (2021). DNA sequences and associated metadata have been deposited in the European Nucleotide Archive (ENA) under accession no. PRJEB39191.

### Data Analysis

Demultiplexing the output data from Illumina sequencing was performed with BCL2FASTQ2 software (Illumina, ver. 2.20.0.422), and primers were trimmed by CUTADAPT ver. 1.18 [38]. Subsequently, raw amplicons were analyzed by the DADA2 pipeline into tables of amplicon sequence variants (ASVs) per sample. The detailed parameters used in each step are described in the paper of Novotny et al. [36]. The taxonomy annotation of ASVs was performed by Naïve Bayesian Classifier for rRNA taxonomic assignment within the DADA2 pipeline. A combination of the SlLVA database [39] and the PhytoREF database [40] was used as a reference for the taxonomic assignment of prokaryote and photoautotrophic organisms. ASVs that failed to be annotated were marked with “x” in the corresponding taxonomy level (i.e., Rickettsialesxxx represents an unidentified species under order Rickettsiales). To focus the analysis on heterotrophic bacterial phyla, autotrophic bacteria were excluded based on information from the literature [30, 41–43]. Samples with sequencing yield < 500 bp were discarded.

Downstream data analysis was done in R 4.2.1 [44] with the *phyloseq* 1.40.0 package for data filtration [45]. We filtered and included ASVs that exist in all replicates of the zooplankton and water samples and where each ASV contributed to at least 1% relative abundance in each individual sample. Rarefaction curves indicated that the species number in all retained samples approached a plateau. To identify host and seasonal effects, we performed the PerMANOVA analysis based on the Bray–Curtis distance (the Bray–Curtis dissimilarity among bacterial communities as the response variable and zooplankton hosts and sampling seasons as the explanatory variables). Additionally, the interaction between the zooplankton host and sampling season was tested with Bonferroni-corrected *P*-values using the *vegan* 2.6–4 package [46]. For testing the assumption of dispersion homogeneity, we used the PerMDISP test. To visualize host and seasonal clustering patterns of bacterial composition, we used nonparametric multidimensional scaling (NMDS) with the Bray–Curtis distance. Bacteria host and temporal variation of alpha and beta diversity were calculated as the Shannon index and Bray–Curtis distance based on the relative abundance of ASVs for each sample. To identify key bacteria contributing to temporal variance in each zooplankton, the contribution of each ASV to the Bray–Curtis dissimilarity between bacterial communities was calculated with *simper* function (available in the vegan package), including a permutation process for significance analysis. We selected core ASVs, defined as those occurring throughout the sampling period in each zooplankton host and water. To compare bacterial correlation patterns across zooplankton genera, correlation patterns between core ASVs shared by all zooplankton were calculated across months, using the Kendall correlation test in the R package *stats* with log-transformed relative ASV abundances.

We also used generalized linear models (GLM) with a quasi-Poisson error distribution (log link function) for the correlation of the Shannon index of bacterial communities and the diet components of each zooplankton genus across the entire sampling period. The diet composition was calculated as a selectivity index based on DNA metabarcoding of the major phytoplankton taxonomic groups, including chlorophytes, diatoms, filamentous cyanobacteria, picocyanobacteria, and other phytoplankton as suggested by Novotny et al. [37]. To investigate if the core microbiota of each zooplankton host was influenced by diet, we tested correlations between the Bray–Curtis dissimilarity matrixes of the diet and the gut bacteria using the Mantel test with Spearman’s rank correlation.

## Results

### Bacterial Community Clustering

**Fig. 1:**
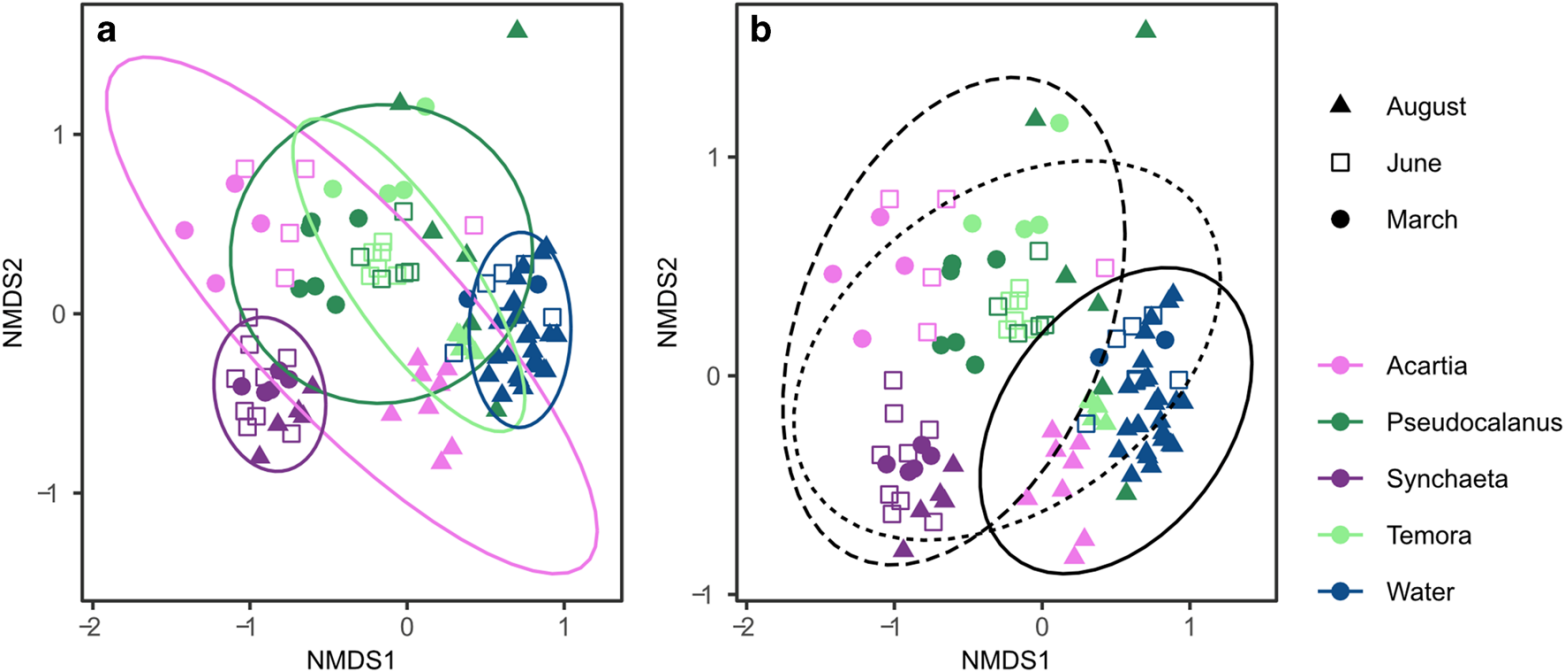
NMDS plots of the bacterial communities across **a** water and zooplankton hosts and **b** months. Zooplankton genera and water are represented with colors, and months with symbols. Ellipses following the *t*-distribution of the NMDS scores are included (March with dashed, June with dotted, and August with solid lines)

A total of 7,281,735 reads with 7878 ASVs was obtained from Illumina sequencing for all water and zooplankton samples. After ASV filtration, we obtained 4,089,094 reads of 143 ASVs belonging to heterotrophic bacteria from both 34 water and 68 zooplankton samples (40,089 ± 26,599 reads/sample). Based on the ASV composition, NMDS ordination suggested that the bacterial communities clustered according to host and month (Fig. 1). The variances of bacterial communities across hosts and months were both significant, yet host differences explained more variance than month (PerMANOVA, Table 1). However, as the dispersion of the bacterial communities differed among zooplankton hosts (Fig. 1a, PerMDISP, *F*_(4,97)_ = 3.09, *P* = 0.019), a non-homogenous variance may interfere with these results. While the cluster dispersion was similar between months (Fig. 1b, F_(2,99)_ = 0.18, *P* = 0.84), the interaction between zooplankton host and sampling season was significant (PerMANOVA, *F*_(8, 87)_ = 2.63, *P* = 0.001). Homogeneous variance of all 14 zooplankton-by-month clusters was found when excluding the *Synchaeta* March sample cluster (PerMDISP, *F*_(13,83)_ = 1.76, *P* = 0.063)), suggesting host-specific temporal variance of the bacterial communities. Tukey’s HSD suggested that *Synchaeta* had the lowest dispersion, indicating less variable bacterial communities compared to the copepods. Despite the heterogeneity of the dispersion, host clustering patterns shown by NMDS ordination supported the importance of host identity for bacterial community composition, as was suggested by PerMANOVA.

**Table 1:** Statistical output of PerMANOVA analysis of bacterial community across the water and host zooplankton samples and months

	Df	Sum of Sqs	*R*^2^	*F*	Pr
Water and Zooplankton host	4	12.22	0.32	12.62	0.001
Month	2	1.79	0.05	3.70	0.001
Residual	95	22.99	0.60		
Total	101	38.42	1		

### Bacterial Diversity Across Water and Zooplankton Hosts

**Fig. 2:**
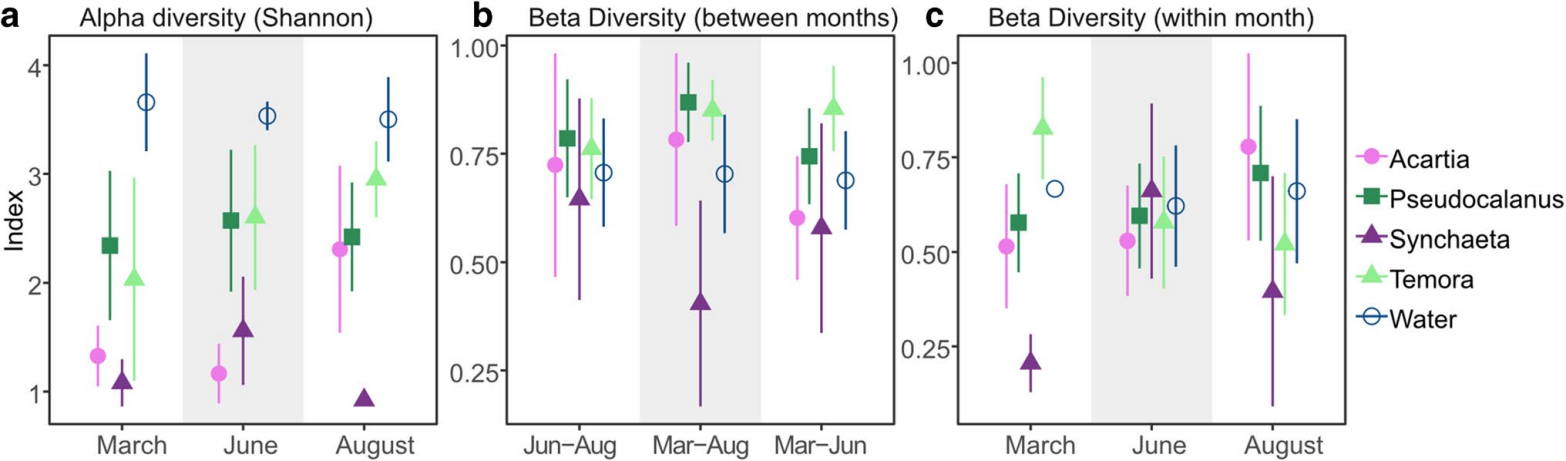
Alpha and beta diversity patterns of bacterial communities. **a** Alpha diversity (Shannon) of water and zooplankton hosts across months. **b** Pairwise beta diversity (the Bray–Curtis dissimilarity) of zooplankton hosts between two sampling months. **c** Beta diversity of water and zooplankton hosts within month. The symbols represent the mean and the error bars standard deviations. Note that the error bar of water in March (**c**) is missing because of only two water samples

The analysis of bacteria diversity showed host-specific temporal dynamics of symbiotic bacteria. Estimates of alpha diversity divided samples into three categories: filtered water, *Temora* and *Pseudocalanus*, and *Acartia* and *Synchaeta* (Fig. 2a). Filtered water samples had the highest diversity (pairwise *t*-test with Bonferroni’s correction, *P* < 0.001), *Temora* and *Pseudocalanus* had similar (*P* = 1.00) and intermediate diversity, whereas *Acartia* and *Synchaeta* had the lowest diversity (*P* < 0.005). Notable is that the diversity of the latter two was higher in August (*Acartia*, *P* = 0.008) and June (*Synchaeta*, *P* = 0.01). High beta diversity (Bray–Curtis index > 0.5) between sampling months of all zooplankton hosts and the water samples, except for a low beta diversity in *Synchaeta* between March and August (*P* < 0.001), indicated shifts in bacterial community over the season (Fig. 2b). Estimates of beta diversity within months suggested variation of the bacterial community between zooplankton individuals, with the lowest variation for *Synchaeta* in March (*P* < 0.001), similar variability for all zooplankton and water samples in June, and less variation for *Temora* and *Synchaeta* in August (*P* < 0.001) (Fig. 2c).

### Bacteria Associated to Zooplankton

**Fig. 3:**
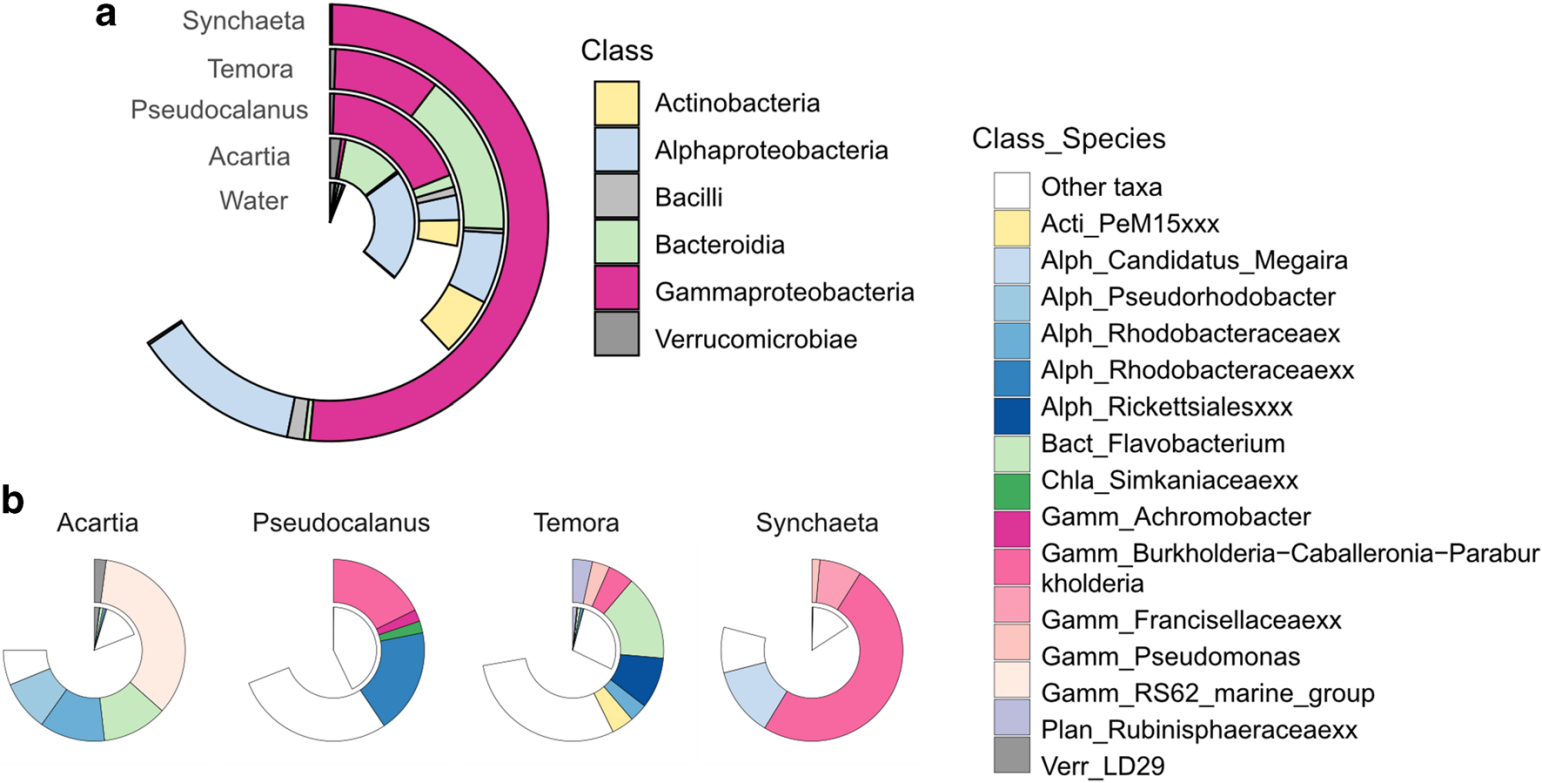
Relative abundance of shared and core ASVs compared to water (inner ring). **a** Relative abundance of shared ASVs present in all zooplankton hosts across months at class level (outer rings) compared to water. **b** Relative abundance of core ASVs across months (outer ring) for each zooplankton host and their relative abundance in water samples (inner ring)

In total, we found 129 core ASVs present throughout the sampling period in all zooplankton hosts and water samples, which represented between 50 and 75% of total bacterial read abundance. However, only 28 core ASVs were shared across all zooplankton hosts, and the shared ASVs were present at low relative abundance in the filtered water (Fig. 3a). These shared core ASVs represented more than 25% of relative bacterial abundance in all zooplankton, and more than 60% in *Synchaeta*, dominated by the classes Gammaproteobacteria, Bacterioida, and Alphaproteobacteria (Fig. 3a). Among the core ASVs in each individual zooplankton host and filtered water samples, water had the highest number (109 ASVs), followed by *Pseudocalanus* (79), *Temora* (71), *Synchaeta* (54), and *Acartia* (44) which corresponds to the higher alpha diversity in *Pseudocalanus* and *Temora* compared with *Synchaeta* and *Acartia* (Fig. 2).

**Fig. 4:**
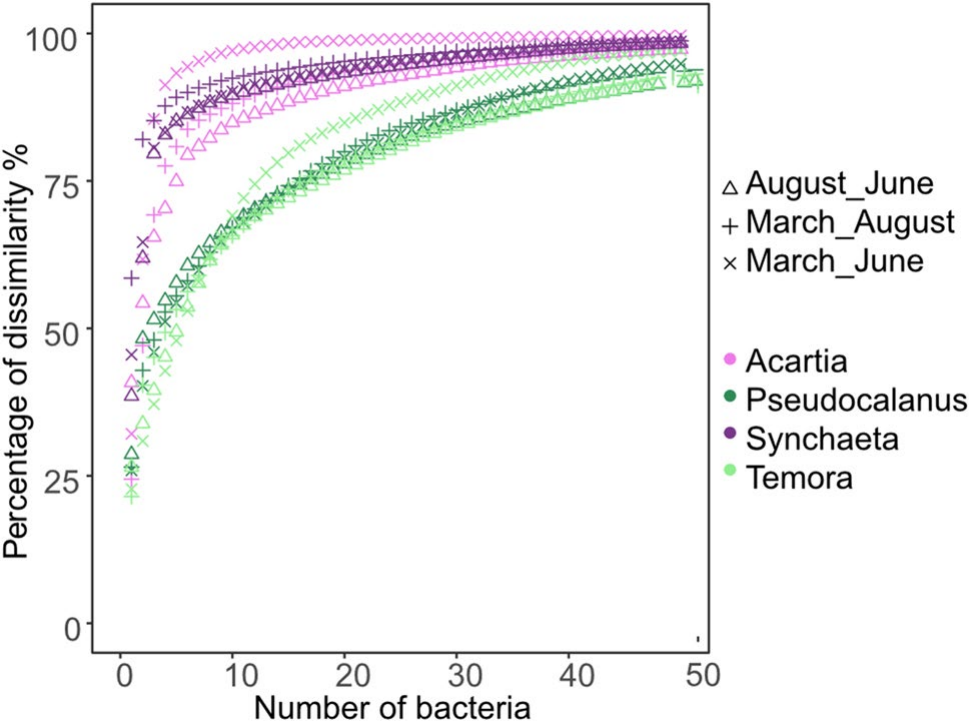
Accumulation curves (the Bray–Curtis dissimilarity) of ASVs for each zooplankton host, indicating the number of ASVs contributing to dissimilarity

Among the 98 core ASVs found in zooplankton hosts, the dominance of certain ASVs showed host-specific distribution patterns (Fig. 3b). *Acartia* was mainly associated to Burkholderiaceae *RS62-marine group* (ASV 2, 35% of total relative abundance ± 26 SD), Flavobacterium (ASV 3, 12% ± 19 SD), and an unknown species of Rhodobacteraceae (ASV 16, 12% ± 14 SD); *Pseudocalanus* to an unknown species of Rhodobacteraceae (ASV 14, 18% ± 21 SD) and Burkholderia-Caballeronia-Paraburkholderia (ASV 1, 19% ± 21 SD); and *Temora* to Flavobacterium ASV 3 (15% ± 18 SD), whereas *Synchaeta* was mainly associated to Burkholderia-Caballeronia-Paraburkholderia (ASV 1, 50% ± 27 SD) and *Candidatus megaira* (ASV 13, 12% ± 11SD) (Table 2). These dominating core ASVs significantly contributed to the variance of bacterial communities between zooplankton genera, in addition to a few less dominant ASVs in specific zooplankton (Simper analysis, Table S1), suggesting that the less dominating bacteria existed across zooplankton genera were also host-specific. About 75% of the temporal gut microbiota dissimilarity in each zooplankton host can be explained by less than five ASVs in *Acartia* and *Synchaeta*, while for *Temora* and *Pseudocalanus* over 10 ASVs are required to explain bacterial community dynamics (Fig. 4), suggesting that the bacteria have more dominating effects on the variance between season in *Acartia* and *Synchaeta*.

**Table 2:** Abundance summary of dominating core ASVs in each zooplankton across sampling months

Host	Bacteria	Mean	SD
*Temora*	Flavobacterium (ASV 3)	15%	18%
*Pseudocalanus*	Burkholderia-Caballeronia-Paraburkholnderia (ASV 1) Rhodobacteraceaexx (ASV 14)	19% 18%	21% 21%
*Acartia*	Burkholderiaceae RS62-marine group (ASV 2) Flavobacterium (ASV 3) Rhodobacteraceaex (ASV 16) Pseudorhodobacter (ASV 12)	35% 12% 12% 9%	26% 19% 14% 9%
*Synchaeta*	Burkholderia-Caballeronia-Paraburkholnderia (ASV 1) Candidatus Megaira (ASV 13)	50% 12%	27% 11%

### Correlation Within Bacterial Microbiota

**Fig. 5:**
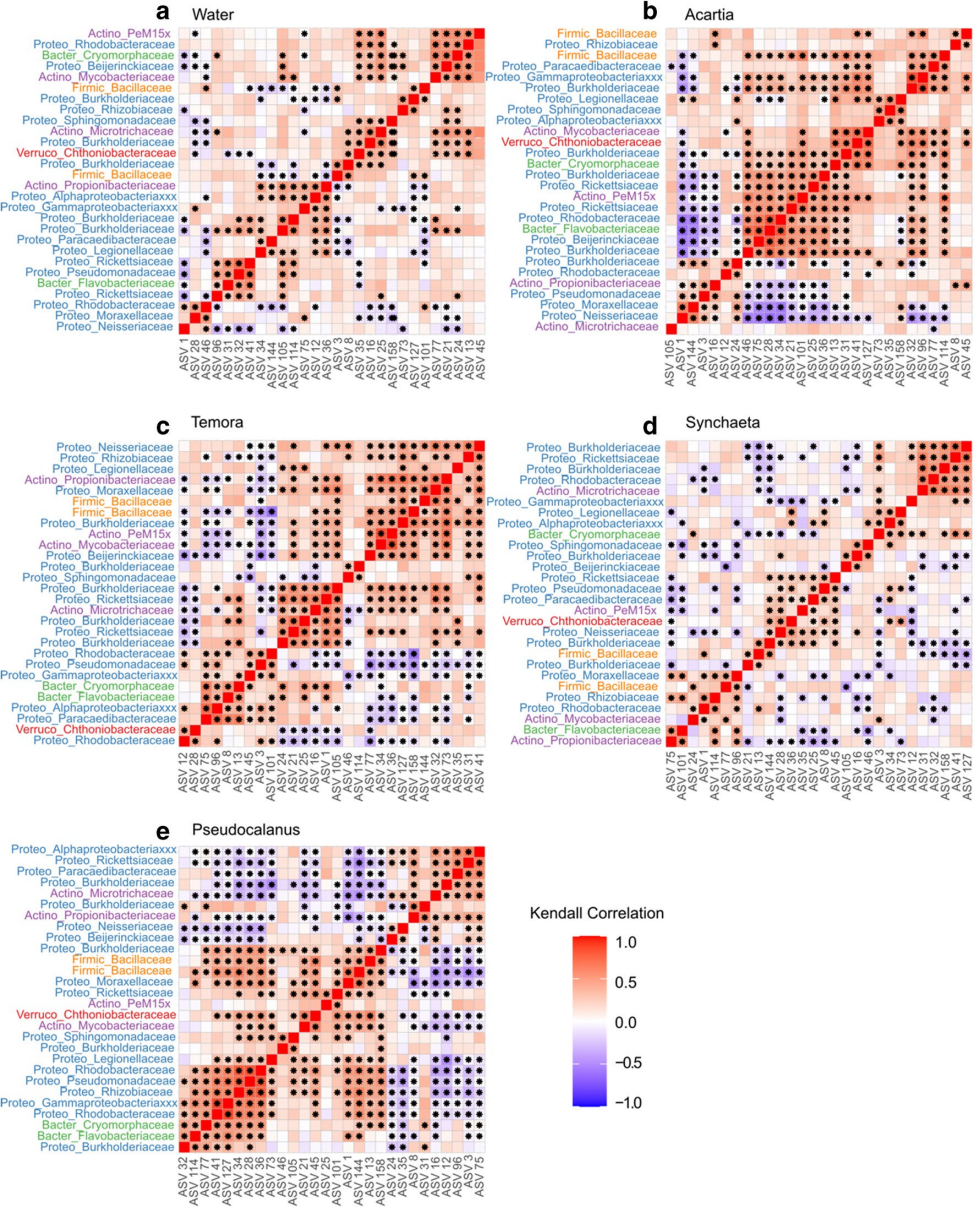
Kendall’s rank correlation coefficients of 28 core bacteria in **a** water, **b** *Acartia*, **c** *Temora*, **d** *Synchaeta*, and **e** *Pseudocalanus*. Correlations are based on the log-transformed relative abundance of ASVs. Asterisks indicate a significant correlation at *P* < 0.05. *Y*-axis labels are the combination of Phylum abbreviates and Family names of the corresponding ASVs on the *x*-axis. The ASVs were ordered hierarchically based on the correlation coefficient

Correlation patterns between the shared 28 core ASVs within zooplankton hosts across the season showed different patterns between zooplankton hosts (Fig. 5). In *Acartia* and *Pseudocalanus*, the bacteria were divided into two groups that were negatively correlated, suggesting co-occurrence within groups and mutual exclusion between groups. Co-occurrence or positive correlations include ASVs belonging to Proteobacteria, Actinobacteria, Firmicutes, Verrucomicrobia, *Acartia*, and Bacteroidetes. In *Temora*, positive correlations across ASVs were similarly observed but with less distinctive groups, while few significant bacterial correlations were observed in *Synchaeta* (Fig. 5). The correlation patterns indicate that each zooplankton host had unique gut bacterial interactions and that the correlation pattern of *Synchaeta* was more similar to the water samples, suggesting weak host regulation.

### Correlation Between Bacterial Alpha Diversity and Diet Composition

Zooplankton diets consisting of diatoms and filamentous cyanobacteria were negatively correlated with the alpha bacteria diversity in all zooplankton hosts (Table 3). Yet, the Mantel test suggested that diet differences never explained the dissimilarity of gut bacteria composition between zooplankton hosts (*r* =  − 0.02, *P* = 0.75). These results suggest that the zooplankton diet affects the diversity of the gut bacterial communities but not the specific succession outcome of bacterial communities’ composition.

**Table 3:** Statistical output of correlation between bacterial communities and diet composition across all zooplankton genera. Output of GLM analysis of each diet component correlating with alpha diversity of gut bacteria

	Estimate	Std. error	*t*-value	Pr( >|*t*|)
(Intercept)	0.98	0.19	5.3	< 0.001
Chlorophytes	− 0.41	0.24	− 1.76	0.08
Diatom	− 0.81	0.28	− 2.95	0.004
Filamentous cyanobacteria	− 0.54	0.26	− 2.09	0.04
Picocyanobacteria	0.78	0.57	1.36	0.18
Other phytoplankton	0.09	0.29	0.31	0.76

## Discussion

Invertebrates like zooplankton are expected to develop less stable gut conditions and more strongly fluctuating gut bacterial communities compared to mammals [22, 28]. Yet, in this study, host differences showed a stronger effect than seasonal differences on the gut bacterial community. Despite that distinct gut bacterial community compositions were found in different zooplankton hosts, compositional clustering patterns, temporal changes of diversity, and bacteria contributing to the temporal variance in the gut communities detected resemblances among the four zooplankton genera that were inconsistent with the taxonomic relations of the hosts. Distinct symbiotic bacteria distribution patterns in zooplankton indicate the importance of host-related traits in shaping gut bacteria composition.

### Gut Bacteria Dynamics of Temora and Pseudocalanus With High Diversity

Clustering patterns suggest different bacterial compositions among the zooplankton, but also several similarities between particularly *Temora* and *Pseudocalanus*. These two copepod genera had a higher alpha diversity and more bacteria species contributing to the temporal variance than did *Synchaeta* and *Acartia* (Figs. 2B and 4 and Table 2). Higher diversity and temporal variance of *Temora* and *Pseudocalanus* are possibly related to their deeper distribution in the water column and their vertical migration behavior [36, 47], exposing the zooplankton to a wider range of bacterioplankton across the different water layers [48]. In addition to their vertical migration behavior, the resemblance of their bacteria diversity can potentially be explained by a similar feeding behavior, although the dominating core bacteria and community composition differed between *Temora* and *Pseudocalanus*. Both genera had a diet composed of Cyanobiaceae and Chlorophyta during March [36], which may establish similar gut environments of the zooplankton during early life stages, eventually leading to the observed bacterial communities in the adults. The importance of bacterial inoculation at the early life stages with long-term effects was previously documented in squid larvae and human infants [22, 34], and may be a common phenomenon.

### Acartia and Synchaeta Had Less Diverse Community With Less Bacteria Contributing to the Temporal Variance

*Acartia* and *Synchaeta* show less vertical migration and are mainly present in the upper 30 m water column, above the thermocline depth [36]. This more constrained vertical distribution probably exposes *Acartia* and *Synchaeta* to stable bacteria seed banks for gut bacteria inoculation because earlier studies suggest that the distribution and diversity of bacterioplankton are significantly related to water-column stratification [49]. *Acartia* had in comparison with the other copepods an unexpectedly low diversity of gut bacteria and fewer bacteria contributing to the temporal variation of gut communities, although the bacteria diversity increased during August. Higher diversity in August may be related to the presence of different *Acartia* species, while *A. tonsa* mainly dominates during the early season [50]. Instead of other copepods, *Acartia* more resembled the rotifer, *Synchaeta*. The diet analysis also showed that *Acartia* and *Synchaeta* shared a diet with high proportions of filamentous cyanobacteria, which may have contributed to the low bacteria diversity and fewer bacteria contributing to the temporal variance in their gut communities [36, 51]. This effect of diet components on gut bacteria is supported by the negative correlation between diatom and filamentous cyanobacteria with gut microbiota diversity for the entire zooplankton community. Filamentous cyanobacteria potentially played a crucial role in shaping gut microbiome in zooplankton in *Acartia* and *Synchaeta*. Mantel’s tests however showed that the similar gut bacteria composition was unrelated to the diet similarity. These findings suggest a general influence from the diet on niche structure, as indicated by diversity and number of key bacteria, rather than effects on specific bacteria composition in zooplankton gut from field studies. This conclusion is in contrast to the expected direct influence of diet phytoplankton on specific gut bacteria composition as observed in laboratory studies [20, 23, 35] In nature, diverse bacteria with redundant functionality from ambient environments may fill niches in the zooplankton gut and interfere with the correlation between diet and specific bacteria composition [16, 52].

### Correlation Patterns Reflected Host Genus-Related Regulations

Gut bacteria correlation patterns distinguished the rotifer from the copepods. Two groups of bacteria with within-group positive correlations were generally found in the copepod hosts with negative correlations between these bacteria groups. This correlation pattern suggests a combination of competition and antagonism along with mutualistic interactions in copepod guts, agreeing with research on mammalian gut bacteria [1, 17, 32]. Notably, our correlation results were based on the seasonal change of abundance, and it is debatable how important bacterial interactions and random processes are for the fluctuations. The suggested importance of bacteria interactions, nevertheless, indicates that further studies on the functional annotation of co-occurring bacteria are needed, and the correlation patterns suggest two alternative gut symbiont regimes in copepods during the sampling seasons.

The core bacteria ASVs were less grouped over time in *Synchaeta* whose correlation pattern was instead closer to the pattern in water. This similarity suggests a strong environmental influence on the bacterial community, but this conclusion is contradicted by the low clustering dispersion and low beta diversity in *Synchaeta* which instead suggest a higher stability of gut bacteria over the season. The dynamics of gut bacteria in *Synchaeta* can also be seen as contradictory with general ecology theory which suggests that stable communities require a diverse community with a high level of interactions [16, 17, 29, 34, 53]. The bacterial community pattern of *Synchaeta* suggests unique regulation of rotifer gut symbionts, probably from specific regulation related to the host species. The host-dependent gut microbiota has been found in zooplankton in the laboratory, and multiple underlying mechanisms were proposed, such as the response of the host immune system, or other physical or chemical host screen mechanisms favoring specific dominating bacteria [1, 5, 19, 54]. The dominating bacteria of *Synchaeta*, Burkholderia-Caballeronia-Paraburkholderia, widely exists in invertebrates, mammals, and plants [55, 56] and includes species with different pathogenic or symbiotic capacities. Burkholderia-Caballeronia-Paraburkholderia was also found in *Pseudocalanus*; in this case, in combination with diverse and correlated bacterial communities, but whether the same strains of Burkholderia-Caballeronia-Paraburkholderia are present in *Synchaeta* and *Pseudocalanus* remains to be verified.

In conclusion, we find that the gut microbiome of zooplankton showed host-specific clustering patterns. Each host genus had unique bacteria contributing to the temporal variability of gut communities, and the temporal succession of gut microbiota was also different between genera. Each of the copepods had two bacteria groups with positive within-group correlations, while the rotifer had less correlated bacterial communities that more resembled the ambient water. Temporal variance of bacterial composition and bacterial correlation showed different similarities of gut bacteria patterns of zooplankton, which may be related to the ecological niche and taxonomy of the hosts. To further investigate the distribution of gut bacteria associated with zooplankton, the next step would be to classify the bacteria into functional groups in order to examine distinct bacteria with redundant functions. For better elucidation of underlying mechanisms of bacteria dynamics and host taxonomy, transcriptomic and proteomic analysis can be further implemented on the entire holobiont, drawing a functional network of bacteria and host metabolism. Overall, our results from this field study suggest that gut bacteria dynamics are not just related to host taxonomy but are also affected by host behavior and life history traits, such as feeding patterns.

### Supplementary Information

Below is the link to the electronic supplementary material.

Supplementary file1 (DOCX 27 KB)

## Data Availability

The original dataset is available in the European Nucleotide Archive (ENA) under accession no. PRJEB39191. The datasets generated during and/or analyzed during the current study are available from the corresponding authors on reasonable request.
