# Supplementary material for: Dynamics of Gut Bacteria Across Different Zooplankton Genera in the Baltic Sea
Source: Microb Ecol. 2024 Feb 26;87:48. doi: 10.1007/s00248-024-02362-7 (PMC10896951; doi:10.1007/s00248-024-02362-7)
Supplement: Supplementary file 1 [file 248_2024_2362_MOESM1_ESM.docx]

**Supplementary Information**

Table S1: ASV in each zooplankton and their contribution to the dissimilarity of pairwise comparison from SIMPER analysis. These ASVs were selected by ranking ASV according to their contribution to the variation (average). The first ASVs whose accumulated dissimilarity (cusum) reach 75% were kept. These ASVs also need to have P<0.05 in the permutation test.

| Zooplankton_pair | ASV id | average | sd | ratio | ava | avb | p | cusum |
| --- | --- | --- | --- | --- | --- | --- | --- | --- |
| *Acartia_Pseudocalanus* | Burkholderiaceae RS62-marine-group (ASV 2) | 0.20 | 0.15 | 1.39 | 0.35 | 0.00 | 0.001 | 0.22 |
|  | Rhodobacteraceaexx (ASV 14) | 0.12 | 0.12 | 0.95 | 0.00 | 0.19 | 0.001 | 0.35 |
|  | Rhodobacteraceaex (ASV 16) | 0.07 | 0.08 | 0.86 | 0.12 | 0.02 | 0.001 | 0.62 |
|  | Pseudorhodobacter (ASV 12) | 0.05 | 0.05 | 1.01 | 0.09 | 0.01 | 0.001 | 0.67 |
|  | Chryseobacterium (ASV 66) | 0.02 | 0.05 | 0.40 | 0.02 | 0.00 | 0.038 | 0.69 |
|  | Achromobacter (ASV 24) | 0.01 | 0.02 | 0.69 | 0.00 | 0.02 | 0.001 | 0.73 |
|  | Simkaniaceaexx (ASV 63) | 0.01 | 0.02 | 0.76 | 0.00 | 0.02 | 0.001 | 0.74 |
| *Acartia_Temora* | Burkholderiaceae RS62-marine-group (ASV 2) | 0.20 | 0.14 | 1.39 | 0.35 | 0.00 | 0.001 | 0.24 |
|  | Flavobacterium (ASV 3) | 0.11 | 0.12 | 0.96 | 0.12 | 0.15 | 0.001 | 0.37 |
|  | Rhodobacteraceaex (ASV 16) | 0.06 | 0.07 | 0.89 | 0.12 | 0.03 | 0.002 | 0.45 |
|  | Rickettsialesxxx (ASV 4) | 0.05 | 0.13 | 0.40 | 0.00 | 0.09 | 0.016 | 0.51 |
|  | Pseudorhodobacter (ASV 12) | 0.05 | 0.05 | 1.06 | 0.09 | 0.03 | 0.001 | 0.57 |
|  | Actinobacteria-PeM15xxx (ASV 36) | 0.03 | 0.03 | 0.92 | 0.00 | 0.04 | 0.003 | 0.63 |
|  | Rubinisphaeraceaexx (ASV 26) | 0.02 | 0.03 | 0.87 | 0.00 | 0.03 | 0.007 | 0.66 |
|  | Chryseobacterium (ASV 66) | 0.02 | 0.05 | 0.44 | 0.02 | 0.01 | 0.024 | 0.68 |
|  | Halieaceae-OM60(NOR5) -clade (ASV 19) | 0.02 | 0.02 | 1.13 | 0.00 | 0.03 | 0.001 | 0.73 |
|  | Verticia (ASV 21) | 0.01 | 0.03 | 0.47 | 0.00 | 0.02 | 0.023 | 0.76 |
| *Pseudocalanus_Temora* | Rhodobacteraceaexx (ASV 14) | 0.12 | 0.12 | 0.96 | 0.19 | 0.00 | 0.001 | 0.15 |
|  | Flavobacterium (ASV 3) | 0.10 | 0.11 | 0.87 | 0.02 | 0.15 | 0.005 | 0.39 |
|  | Rickettsialesxxx (ASV 4) | 0.05 | 0.13 | 0.40 | 0.00 | 0.09 | 0.015 | 0.45 |
|  | Actinobacteria-PeM15xxx (ASV 36) | 0.03 | 0.03 | 1.05 | 0.02 | 0.04 | 0.001 | 0.49 |
|  | Rubinisphaeraceaexx (ASV 26) | 0.02 | 0.02 | 0.87 | 0.01 | 0.03 | 0.008 | 0.54 |
|  | Halieaceae-OM60(NOR5)-clade (ASV 19) | 0.02 | 0.01 | 1.14 | 0.01 | 0.03 | 0.001 | 0.60 |
|  | Verticia (ASV 21) | 0.02 | 0.03 | 0.53 | 0.01 | 0.02 | 0.002 | 0.62 |
|  | Achromobacter (ASV 24) | 0.01 | 0.02 | 0.82 | 0.02 | 0.01 | 0.001 | 0.64 |
|  | Simkaniaceaexx (ASV 63) | 0.01 | 0.02 | 0.76 | 0.02 | 0.00 | 0.001 | 0.66 |
|  | Mycobacterium (ASV 34) | 0.01 | 0.01 | 0.99 | 0.01 | 0.01 | 0.002 | 0.67 |
|  | Terrimicrobium (ASV 67) | 0.01 | 0.02 | 0.67 | 0.01 | 0.01 | 0.002 | 0.70 |
|  | Reyranella (ASV 57) | 0.01 | 0.01 | 0.80 | 0.01 | 0.01 | 0.002 | 0.72 |
|  | Rheinheimera (ASV 82) | 0.01 | 0.01 | 0.75 | 0.01 | 0.00 | 0.006 | 0.74 |
| *Synchaeta _Acartia* | Burkholderia-Caballeronia-Paraburkholderia (ASV 1) | 0.30 | 0.16 | 1.82 | 0.50 | 0.01 | 0.001 | 0.31 |
|  | Burkholderiaceae RS62-marine-group (ASV 2) | 0.20 | 0.15 | 1.38 | 0.00 | 0.35 | 0.001 | 0.52 |
|  | Candidatus Megaira (ASV 13) | 0.07 | 0.06 | 1.17 | 0.12 | 0.01 | 0.001 | 0.60 |
|  | Rhodobacteraceaex (ASV 16) | 0.07 | 0.08 | 0.85 | 0.00 | 0.12 | 0.001 | 0.67 |
|  | Pseudorhodobacter (ASV 12) | 0.05 | 0.05 | 0.98 | 0.00 | 0.09 | 0.001 | 0.79 |
| *Synchaeta_Pseudocalanus* | Burkholderia-Caballeronia-Paraburkholderia (ASV 1) | 0.25 | 0.15 | 1.67 | 0.50 | 0.18 | 0.001 | 0.32 |
|  | Rhodobacteraceaexx (ASV 14) | 0.12 | 0.12 | 0.96 | 0.00 | 0.19 | 0.001 | 0.47 |
|  | Candidatus Megaira (ASV 13) | 0.08 | 0.06 | 1.21 | 0.12 | 0.01 | 0.001 | 0.57 |
|  | Francisellaceaexx (ASV 20) | 0.05 | 0.10 | 0.53 | 0.07 | 0.00 | 0.001 | 0.63 |
|  | Achromobacter (ASV 24) | 0.01 | 0.02 | 0.70 | 0.00 | 0.02 | 0.001 | 0.65 |
|  | Simkaniaceaexx (ASV 63) | 0.01 | 0.02 | 0.76 | 0.00 | 0.02 | 0.001 | 0.67 |
|  | Anaerobacillus (ASV 41) | 0.01 | 0.01 | 0.96 | 0.01 | 0.01 | 0.016 | 0.74 |
| *Synchaeta_Temora* | Burkholderia-Caballeronia-Paraburkholderia (ASV 1) | 0.28 | 0.14 | 2.01 | 0.50 | 0.05 | 0.001 | 0.31 |
|  | Flavobacterium (ASV 3) | 0.10 | 0.11 | 0.85 | 0.00 | 0.15 | 0.008 | 0.42 |
|  | Candidatus Megaira (ASV 13) | 0.08 | 0.06 | 1.21 | 0.12 | 0.00 | 0.001 | 0.50 |
|  | Rickettsialesxxx (ASV 4) | 0.05 | 0.13 | 0.40 | 0.00 | 0.09 | 0.016 | 0.56 |
|  | Francisellaceaexx (ASV 20) | 0.05 | 0.09 | 0.53 | 0.07 | 0.00 | 0.010 | 0.61 |
|  | Actinobacteria-PeM15xxx (ASV 36) | 0.03 | 0.03 | 0.92 | 0.00 | 0.04 | 0.001 | 0.64 |
|  | Pseudomonas (ASV 8) | 0.02 | 0.06 | 0.39 | 0.01 | 0.03 | 0.031 | 0.67 |
|  | Rubinisphaeraceaexx (ASV 26) | 0.02 | 0.03 | 0.88 | 0.00 | 0.03 | 0.005 | 0.70 |
|  | Halieaceae-OM60(NOR5)-clade (ASV 19) | 0.02 | 0.02 | 1.14 | 0.00 | 0.03 | 0.001 | 0.74 |
